# A Nanostructured Sensor Based on Gold Nanoparticles and Nafion for Determination of Uric Acid

**DOI:** 10.3390/bios8010021

**Published:** 2018-03-06

**Authors:** Natalia Stozhko, Maria Bukharinova, Leonid Galperin, Khiena Brainina

**Affiliations:** 1 Ural State University of Economics, 8 Marta St., 62, Yekaterinburg 620144, Russia; sny@usue.ru (N.S.); mbuharinova@mail.ru (M.B.); 2 Ural Federal University, Mira St., 19, Yekaterinburg 620002, Russia; lhalp@k66.ru

**Keywords:** uric acid, carbon screen-printed electrode, Nafion, gold nanoparticles, nanoeffects, ”green” synthesis, milk, blood serum

## Abstract

The paper discusses the mechanism of uric acid (UA) electrooxidation occurring on the surface of gold nanoparticles. It has been shown that the electrode process is purely electrochemical, uncomplicated with catalytic stages. The nanoeffects observed as the reduction of overvoltage and increased current of UA oxidation have been described. These nanoeffects are determined by the size of particles and do not depend on the method of particle preparation (citrate and “green” synthesis). The findings of these studies have been used to select a modifier for carbon screen-printed electrode (CSPE). It has been stated that CSPE modified with gold nanoparticles (5 nm) and 2.5% Nafion (Nf) may serve as non-enzymatic sensor for UA determination. The combination of the properties of nanoparticles and Nafion as a molecular sieve at the selected pH 5 phosphate buffer solution has significantly improved the resolution of the sensor compared to unmodified CSPE. A nanostructured sensor has demonstrated good selectivity in determining UA in the presence of ascorbic acid. The detection limit of UA is 0.25 μM. A linear calibration curve has been obtained over a range of 0.5–600 μM. The 2.5%Nf/Au(5nm)/CSPE has been successfully applied to determining UA in blood serum and milk samples. The accuracy and reliability of the obtained results have been confirmed by a good correlation with the enzymatic spectrophotometric analysis (R^2^ = 0.9938) and the “added−found” technique (recovery close to 100%).

## 1. Introduction

Uric acid (UA) plays a dual role in the human body: on the one hand, it exhibits antioxidant properties; on the other hand, it causes formation of urates in the tissues in case of purine metabolic disorder. Abnormalities of UA levels are symptoms of Lesch–Nyhan syndrome, multiple sclerosis, cardiovascular, and renal diseases [1,2]. In this regard, monitoring of UA is essential for clinical diagnostics and food analysis.

To determine UA, electrochemical methods are applied. Electrochemical methods have advantages over other methods, as they are more sensitive, selective and can be easily automated. The methods of electrochemical determination of UA in biological liquids (mainly urine) have been developed, but the methods of electrochemical determination of UA in food stuff have not been dealt with so far. Modified glassy carbon electrodes (GCE) are most frequently used for UA determination [3,4,5,6,7,8,9,10]. Its modifying layers can include gold nanoparticles [3,5,6,7,8,9,10], nanotubes [5,6], reduced graphene oxide [3,7], chitosan [7], Nafion and polymers [4,8], cyclodextrins [9], or their combinations. There are cases of using screen-printed electrodes, modified with single and multi-wall carbon nanotubes [11,12,13], chemically-reduced graphene oxide [14,15], nanoparticles of noble metals [16], or core-shell nanocomposites [17], and enzymes [18,19], in order to determine UA in biological fluids. Unlike GCE, a planar design of screen-printed electrodes enables to use them in portable devices for on-site analysis. Screen-printed electrodes are cheap, as they are manufactured with the use of the technology that provides mass production of transducers with good reproducible characteristics [20]. Significant weaknesses of the exiting modified GCE and screen-printed electrodes, used for UA determination are the following:

- necessity of GCEs surface pretreatment before use;
- time consuming (from several hours [14,19] to several days [12,17]) procedures of preparing the modifier and modification of the electrode [5,9,10];
- inability to use GCEs in portable devices designed for on-site analysis;
- short lifetime of enzymes, often requiring special storage conditions;
- necessity to preconcentrate analyte (in some cases) [11,12,13,16,18];
- the use of complex, expensive, non-transportable, malfunctioning flow-injection systems that are difficult to use for on-site analysis [16,18];
- lack of methods of UA electrochemical determination in food products (e.g., milk).

In this regard, it is important to develop a simple enzyme-free screen-printed electrode that does not require any additional operations of separation and concentration, but can be used to determine UA in foods (e.g., milk) and biological objects with a complex matrix, such as blood serum, as the level of UA in blood serum is an essential diagnostic indicator of some diseases. 

In our opinion, a practical approach to developing a sensitive and selective UA sensor is the modification of the screen-printed electrode with gold nanoparticles and electrically-conductive perfluorinated membrane (Nafion), which includes a hydrophobic matrix and hydrophilic pores and channels. The reduction of electrode overvoltage and the increase in oxidation currents of compounds diffusing from the solution on the surface of gold nanoparticles [21], together with the ability of Nafion to act as a molecular sieve in multicomponent media, will ensure high analytical and metrological characteristics of the sensor. The combination of nanoparticles and polymer is very practical, as this reduces agglomeration and recrystallization of nanoparticles and results in their fixing on the electrode surface, thereby increasing the stability of the sensor. Selection of pH medium will improve the resolution sensibility of the sensor.

However, another fundamental issue to be considered in this work is to establish the mechanism of UA electrooxidation on gold nanoparticles. This is due to the existing contradictory views on this issue and to the fact that the earlier proposed approaches and sensors are based on empirical regularities of the processes only. The choice of the modifier and conditions for UA determination should utilize the knowledge of electrode process mechanisms. 

The aims of this study are (i) to investigate the type of electrode process for UA oxidation on the nanostructured electrode; (ii) to specify conditions for selective determination of UA in the presence of AA; (iii) to develop a sensor based on carbon screen-printed electrode (CSPE) modified with gold nanoparticles and Nafion, which could be used in portable devices, and (iv) to develop a method of UA determination in blood serum and milk.

Mechanism of Uric Acid Oxidation on a Nanoparticle-Modified Electrode

Some publications look upon UA electrooxidation as an electrocatalytic process, whereby carbon nanotubes/nanoparticle hybrid material [5], restored graphene oxide [14], β-cyclodextrin [15], and gold nanoparticles [7,16] can act as catalysts. 

There are two possible mechanisms for UA electrooxidation:

1. an electrochemical process, without intermediate chemical stages;
2. an electrochemical process, including the catalytic stage, where the formation and decay of intermediate products is expected.

The interpretation of the process taking place on gold nanoparticles as a process involving the catalytic stage might be problematic.

In order to develop an optimal method of electrochemical determination of UA it may be useful to know the mechanism of the proceeding process. To answer this question, we have used the earlier proposed approach and mathematical model of electrochemical processes, not complicated by chemical stages, but including catalytic stages and nanoeffects [21,22], and have conducted experimental studies.

The mathematical model described in [21,22] was used, which considered electrode processes of various types and nanoeffects. The calculations (results are given below) were performed for the following electrode processes: electrochemical, uncomplicated by other stages (E); electrochemical, including catalytic (EC) stage; and processes with nanoeffects. Equation (1) serves as the basis for these considerations:

(1) $$i=nFk_{S}SC_{S}\exp\{\frac{\beta nF(E_{\mathrm{in}}-E^{\circ }+vt)+\beta \Delta G^{\circ }}{RT}\}$$

where

- *n*, number of electrons involved in the process;
- *F*, Faraday constant (96,485 C mol^−1^);
- *β*, electron transfer coefficient in the anodic phase (0.49 [23]);
- *k*_s_, electrode process rate constant(1.00 × 10^−4^ cm s^−1^), was determined as described earlier [24], using voltammogram of UA oxidation at unmodified GCE;
- *S*, electrode area (cm^2^);
- *C*_s_, surface concentration of electroactive substance diffusing to the electrode (mol cm^−3^);
- *E*_in_, initial potential (0.20 V);
- *E*°, redox potential of UA, is given (relative to Ag/AgCl) for red-ox potential in the reaction ^∙^U^2^^−^+e^−^+H^+^→UH^2^^−^ in accordance with [25] (0.37 V);
- *ν*, potential scan rate (V s^−1^);
- *t*, time (s);
- *R*, universal gas constant (8.314 J mol^−1^ K^−1^); and
- *T*, temperature (K).

Gibbs free energy (Δ*G*°) was calculated using Equation (2) taken from [21]:

(2) $$\Delta G^{\circ }=\frac{3\sigma M}{\rho r}$$

where

- *σ*, surface tension of gold at the air borderline at 700 °C (1200 dyn/cm [26]);
- *M*, molar mass of gold (197 g/mol);
- *ρ*, density of gold (19.3 g/cm^−3^); and
- *r*, radius of nanoparticles.

**Figure 1:**
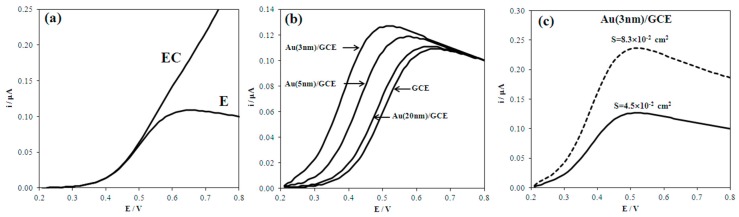
Calculated anodic voltammograms of 0.02 mM UA for different electrode processes: (**a**) electrochemical (E) and electrochemical, including catalytic stage (EC) at GCE; (**b**) electrochemical process at GCE, Au(20 nm)/GCE, Au(5nm)/GCE, Au(3nm)/GCE with equal surface area *S* = 4.5 × 10^−2^ cm^2^; and (**c**) electrochemical process at Au(3nm)/GCE with different nanoparticle surface.

Figure 1a presents the calculated voltammograms of UA oxidation for E and EC processes. It is seen that the shape of the voltammogram, describing the EC process with the catalytic stage, has no region of the limiting current. The shape of this voltammogram differs significantly from the shape of the voltammogram illustrating uncomplicated E process.

Figure 1b,c present the calculated voltammograms that were when the electrode E process included nanoeffects. Nanoeffects are observed as the shift of the voltammograms of the electrodes modified with nanoparticles to the less-positive region, as compared with the unmodified electrode (Figure 1b). The smaller the size of the nanoparticles, greater the nanoeffects. If the surface area of nanoparticles of various sizes is almost the same as the electrode area, then the currents of UA oxidation on these nanoparticles will be commensurable (Figure 1b). Figure 1c illustrates the calculated voltammograms of UA oxidation on gold nanoparticles (3 nm) with different surface areas. In one case, the area of nanoparticles was limited by the size equal to the GCE area (*S* = 4.5 × 10^−2^ cm^2^). In the other case, the real total surface area of nanoparticles (*S* = 8.3 × 10^−2^ cm^2^) was used for the calculations. A significant increase in the UA oxidation current was observed on the nanoparticles with a larger, than the limited area, surface area. Thus, nanoeffects are demonstrated in the decrease in the overvoltage and the increase in the UA oxidation current. 

## 2. Experimental

### 2.1. Chemicals and Apparatus

HCl (NevaReactiv, Saint Petersburg, Russia), HAuCl_4_ (Tom’analit, Tomsk, Russia), sodium citrate (Himreactivsnab, Ufa, Russia), Nafion (Fluka, Buchs, Switzerland), ascorbic acid (Sigma, Shanghai, China), uric acid (Acros Organics, Geel, Belgium), glucose (NevaReactiv, Saint Petersburg, Russia), urea (Fluka, Buchs, Switzerland), L-tryptophan (AppliChem, Darmstadt, Germany), and creatinine (Merc, Darmstadt, Germany) were used. Phosphate buffer solutions (PBS) were used as supporting electrolytes. All chemicals were of extra pure grade and chemically pure grade. 

A semi-automatic computerized voltammetric analyzer IVA-5 (IVA, Yekaterinburg, Russia) with a PE-6100 magnetic stirrer and a three-electrode electrochemical cell was employed for voltammetric measurements. Working electrodes were glassy carbon electrode (GCE) (Metrohm, Herisau, Switzerland), gold disk electrode (Au-bulk) (Metrohm, Herisau, Switzerland) and carbon screen-printed electrode (CSPE) (IVA, Yekaterinburg, Russia). A glassy carbon rod and Ag/AgCl (saturated with KCl) were used as the auxiliary electrode and reference electrode, respectively. 

### 2.2. Synthesis and Characterisation of Gold Nanoparticles

Gold suspension was synthesized by chemical reduction of aqueous solution of chloroauric acid (HAuCl_4_) by sodium citrate (Na_3_C_6_H_5_O_7_) in accordance with the standard Turkevich method and as described in [21]. The gold particles of different sizes were prepared by changing the ratio C(HAuCl_4_):C(Na_3_C_6_H_5_O_7_). Two samples of gold sol Au_red_ and Au_vio_ were obtained with the ratio C(HAuCl_4_):C(Na_3_C_6_H_5_O_7_) equal to 1:5 and 1:2, respectively.

“Green” nanoparticles were obtained using *Hippóphaë* extract. To prepare it, 0.4 g of powdered dry *Hippóphaë* leaves were placed in a beaker, then 10 mL of water was added and mixed for 15 min at room temperature. The solid phase was separated by centrifugation at 10,000 rpm and the liquid phase was used to synthesize nanoparticles.

During “green” synthesis of gold nanoparticles, 250 µL of freshly-prepared *Hippóphaë* extract was added to 5 mL of a boiling solution of 2 × 10^−3^ M HAuCl_4_ and the mixture was intensively stirred.

TEM images of gold sols are given in our previous paper [21]. The gold particles with the radius of 5, 14, and 20 nm prevailed in sols Au_red_, Au_green_, and Au_vio_, respectively.

### 2.3. Preparation of Modified Electrodes

CSPE or GCE as substrates and gold nanoparticles suspensions (5, 14, 20 nm) were used to modify electrodes.

The drop casting method was used to modify CSPE: 5 µL of gold nanoparticles suspension was deposited on the CSPE surface area. Then the electrode was air-dried at room temperature. 

To prepare Nf/Au/CSPE, 10 µL of 0.5, 1.0, 1.5, 2.0, 2.5, and 5.0% Nf were deposited on the Au/CSPE surface area. Then the electrode was air-dried at room temperature. Prior to use, the obtained modified electrode (Nf/Au/CSPE) was washed with distilled water. 

As shown by our SEM studies [21,24], nanoparticles do not aggregate on the electrode surface, so the particle size on the surface was assumed to be the same as in the sol.

### 2.4. Electrochemical Measurements

Linear sweep voltammetry (LSV) was used in ordinary and derivative modes. The derivative curve presents simply differentiated initial voltammogram, dependence d*I*/d*E* = f(*E*) is recorded. 

**Figure 2:**
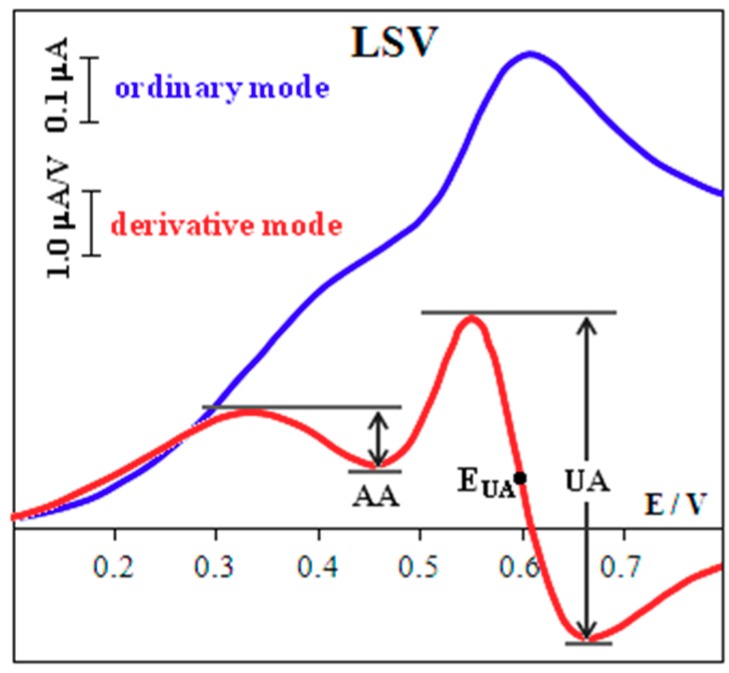
Derivative and ordinary anodic voltammograms of 0.1 mM UA and 0.1 mM AA mixtures at Au(5nm)/CSPE. Background: PBS (рН 5), *ν* = 50 mV/s.

Recording of the anodic voltammograms was carried out using a phosphate buffer (pH 5) at *ν* = 50 mV/s in the range of potential 0.1–0.8 or 0.1–0.9 V. Derivative voltammograms were used due to poor separation of UA and AA signals in LSV. The measured signal is an amplitude of the derivative voltammogram (d*I*/d*E* curve). It is measured as shown in Figure 2. The potential peak corresponds (as mathematical value) to the value of the derivative equal to zero. To exclude background currents the peak current potential is measured as half of the amplitude potential. This value is used for calculating UA and AA signals [27].

### 2.5. Real Samples

Frozen blood serum samples were provided by the clinical laboratory of Medical Technologies JSC, a multidisciplinary medical center. The results were compared with the data obtained in Medical Technologies JSC, via the method of BioSystems (Barcelona, Spain). 

Milk samples from different manufacturers were purchased from local supermarkets and stored at 4 °C until the study. For analysis, aliquots of blood serum (100 μL) or milk (200 μL) were taken, which were introduced into an electrochemical cell containing 10 mL of phosphate buffer solution. Preliminary preparation of blood serum and milk samples was not required.

## 3. Results and Discussion

### 3.1. Comparison of Calculated and Experimental Data 

**Figure 3:**
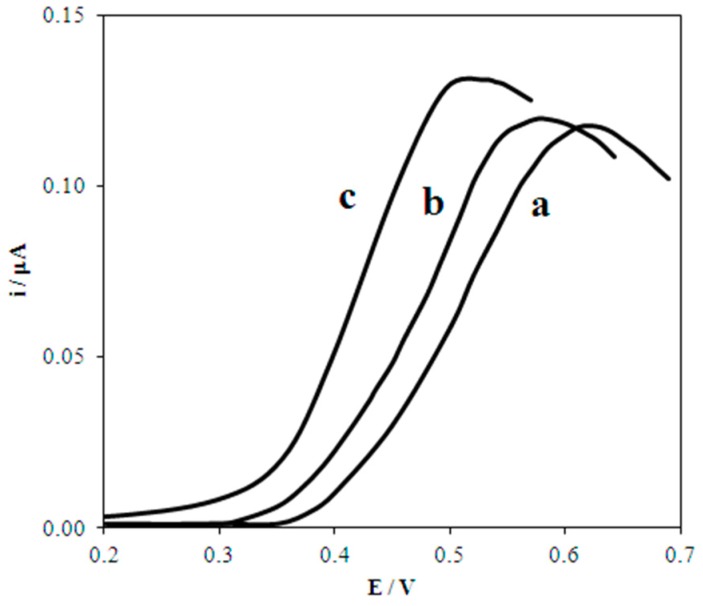
Experimental anodic voltammograms of 0.02 mМ UA at GCE (**a**), Au(20nm)/GCE (**b**) and Au(5nm)/GCE (**c**). Background: PBS (рН 7), *ν* = 50 mV/s.

Figure 3 presents experimental voltammograms of UA oxidation on macro- and nanostructured electrodes. The correspondence of the shape of the experimental curves (Figure 3) and calculated one (Figure 1a) for a process of a certain type shows that a process of this type is taking place. The data presented in Figure 3 illustrates that UA oxidation on macro- and nanostructured electrodes is not complicated by the catalytic stage. The potential shift of UA electrooxidation on the nanostructured electrodes to the cathodic area vs. macroelectrodes testifies to the manifestation of nanoeffects (Table 1).

The smaller the size of the nanoparticles, immobilized on different electrodes, the greater the shift. This shift is caused by an increase in the Gibbs free energy of the nanostructured electrode (Δ*G*° = 7349.22, 1837.31 J mol^−1^ for Au 5, 20 nm, respectively) as compared to the macroelectrode (Δ*G*° = 0 J mol^−1^). These data indicate that nanoeffects in UA electrooxidation on gold nanoparticles are demonstrated. Nanoeffects are observed as a decrease in overvoltage of the electrode process. The effects are greater with a smaller size of nanoparticles, which is due to the contribution of the nanoparticles’ Gibbs free energy to the kinetics of UA electrochemical oxidation. The reason is a lower energy barrier of the reaction [21].

The observed nanoeffects are independent of the nanoparticles synthesis method and the substrate nature. For example, the half-wave potential of UA oxidation on smaller nanoparticles (Au_red_ = 5 nm), obtained by citrate synthesis and immobilized on GCE, shifts by 50 mV to the cathodic area as compared with larger nanoparticles (Au_green_ = 14 nm) obtained by “green” synthesis and immobilized on Au-bulk.

Following the findings of the study, gold nanoparticles Au(5nm) were selected for developing the UA sensor. 

### 3.2. UA Oxidation in the Presence of AA at the CSPE 

**Figure 4:**
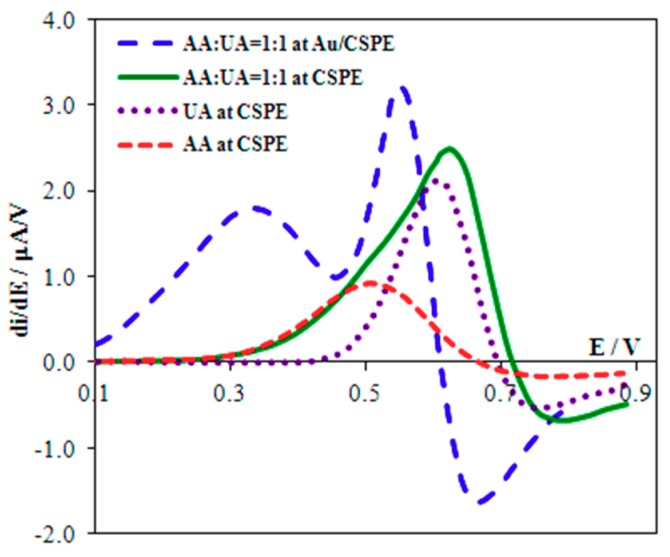
Derivative anodic voltammograms of 0.1 mM UA, 0.1 mM AA at CSPE and their mixture at CSPE and Au(5nm)/CSPE. Background: PBS (pH 5), *ν* = 50 mV/s.

The individual signals of AA and UA oxidation on the CSPE are recorded at the potentials of 0.62 and 0.69 V, respectively, in phosphate buffer solution (pH 5) with a potential difference (Δ*E*) of 0.07 V (Figure 4). In the simultaneous presence of AA and UA their signals overlap and form one broad signal (Figure 4), which increases with growing concentration of both AA and UA.

### 3.3. Separating Signals of UA and AA 

**Figure 5:**
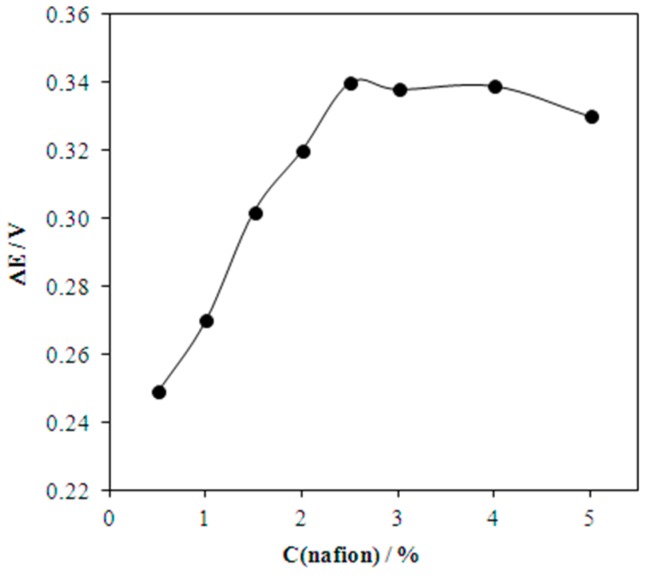
Effect of Nafion concentration on the difference of potentials of 0.1 mM UA and 0.5 mM AA oxidation at the Nf/Au(5nm)/CSPE. Background: PBS (pH 5), *ν* = 50 mV/s.

**Figure 6:**
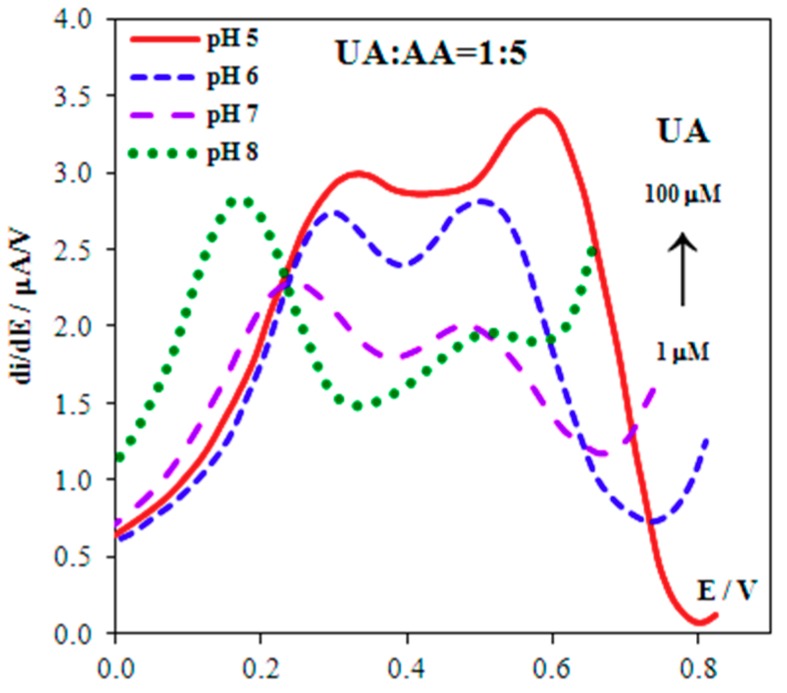
Derivative anodic voltammograms of 0.1 mM UA and 0.5 mM AA at the 2.5% Nf/Au(5nm)/CSPE in background solution with different pH.

The signals of AA and UA, corresponding to the equimolar ratio of their concentrations, are distinguished (Δ*E* = 0.22 V) on derivative voltammograms (Figure 4), and registered using Au(5nm)/CSPE. A five-fold excess of AA is followed by overlapping of the signals. To avoid this phenomenon we protected the surface of the Au(5nm)/CSPE with Nafion. The maximum potential difference of oxidation of AA and UA (Δ*E* = 0.34 V) is observed at 2.5% Nafion concentration (Figure 5) at pH 5. This corresponds to the fact that, at pH 5, the maximum permeability of Nafion for UA and minimum permeability for AA is attained [4]. The data given in Figure 6 shows that these are the best conditions for UA:AA = 1:5 separate signal registration (pH < 5 was not used because of the large background current in the potential range 0.0–0.9 V). In addition, Nafion stabilizes the gold nanoparticles on the electrode surface [28,29].

### 3.4. Analytical Characteristics and Real Samples Analysis

**Figure 7:**
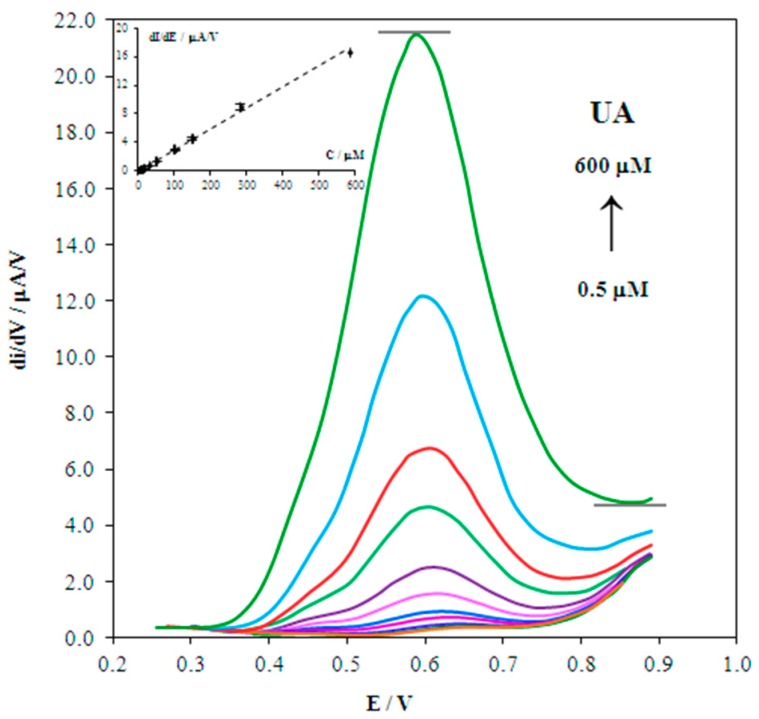
Derivative anodic voltammograms with increasing concentrations of UA (0.5–600 µM) at the 2.5% Nf/Au(5nm)/CSPE. Inset: corresponding calibration curve d*I*/d*E* = f(*C*). Background: PBS (pH 5), *ν* = 50 mV/s.

Figure 7 shows the derivative voltammograms of UA oxidation with different concentrations at the 2.5% Nf/Au(5nm)/CSPE. The magnitude of the UA signal is linearly dependent on UA concentration from 0.5 µM to 600 µM, and is described by the regression equation y = 0.0294x + 0.0359 with a correlation coefficient R^2^ = 0.9966. The detection limit of UA at the 2.5% Nf/Au(5nm)/CSPE is 0.25 μM. The relative standard deviation of the signal for 5 μM UA is 1.9%.

A 100-fold excess of glucose and urea, a 70-fold of creatinine, a 10-fold excess of tryptophan, and a five-fold excess of AA do not interfere with UA determination at the 2.5% Nf/Au(5nm)/CSPE (Table 2).

Analytical characteristics of the 2.5% Nf/Au(5nm)/CSPE remain stable for a month after storage at room temperature.

Analytical characteristics of UA determination with the use of the proposed sensor and other modified CSPEs are displayed in Table 3. The advantages of the developed sensor are as follows: it does not require time-consuming preparatory adsorption concentration [11,12,13] or complex and expensive equipment for FIA [16,18], does not contain rapidly degrading enzymes [18,19], and does not require long preparation of the modifier and modification [14,17]. Unlike most of the known modified CSPE [11,13,14,17,18,19], used in the urine analysis only, the developed sensor enables the analysis of blood serum, and also in a broader linear concentration range than in [15]. Another important advantage of the developed sensor is that it allows us to determine UA in milk without pre-separation and pre-concentration, which none of the existing sensors do.

Table 4 shows the analysis results of blood serum samples. It is apparent from the table that recovery does not exceed 110%, which suggests acceptable reliability of UA determination in blood serum samples obtained using 2.5% Nf/Au(5nm)/CSPE. UA found in all samples of blood serum corresponds to the medical norm (0.15–0.42 mM). 

**Figure 8:**
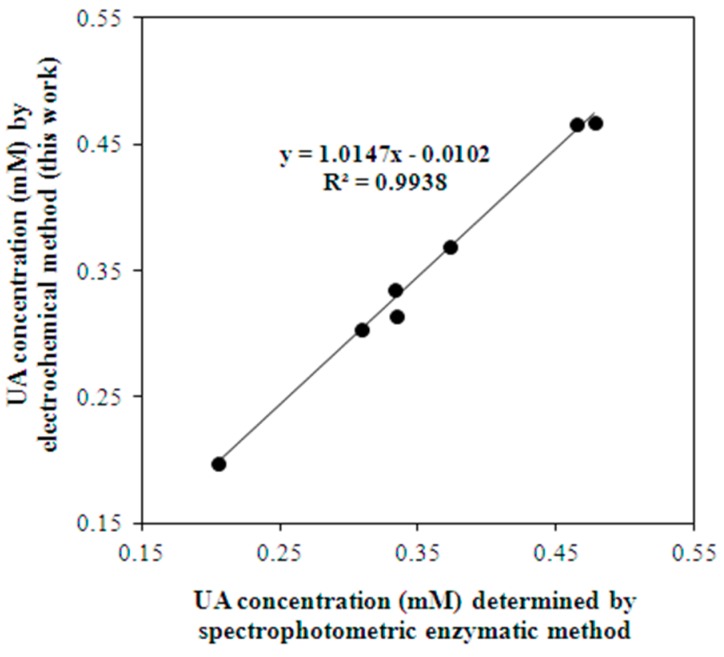
Correlation between results of UA determination in blood serum samples obtained by the enzymatic spectrophotometric method and the proposed electrochemical method.

The comparison of the results of electrochemical UA determination by using 2.5% Nf/Au(5 nm)/CSPE with the results of the enzymatic spectrophotometric method demonstrated a good correlation (R^2^ = 0.993) (Figure 8).

The proposed sensor was tested by adding given amounts of analyte standards into the milk samples. The percentage recoveries were found to be 96.5—101.5% as presented in Table 5. S_r_ does not exceed 2.5%.

## 4. Conclusions

The mathematical modeling and experimental studies have shown that the process of UA electrooxidation with gold nanoparticles is not complicated by catalytic stages. This conclusion proves that the common view that nanoparticles serve as catalysts in electrode processes is not convincing. The data obtained in this paper show that the role of nanoparticles is to contribute the Gibbs free energy to the reaction kinetics.

The nanoeffects of the particles obtained by citrate and “green” synthesis are exhibited in a lower UA oxidation potential, and it is greater with a smaller size of gold nanoparticles. These findings allow us to justify the choice of gold nanoparticles Au (5 nm) for developing an UA sensor. 

It was shown for the first time that the CSPE, modified with 5 nm gold nanoparticles and 2.5% Nafion, and pH 5 background electrolyte ensures selective and sensitive determination of UA in the presence of AA. The combination of the properties of nanoparticles and Nafion as a molecular sieve at the selected pH 5 phosphate buffer solution has significantly improved the resolution of the sensor compared to unmodified CSPE. The developed sensor with a linear range of 0.5–600 μM and a detection limit of 0.25 μM has been successfully applied to determining UA in blood serum and milk samples without prior preparation of the electrode surface, time-taking modification and additional UA concentration, and the use of complex equipment and recording, which allows us to recommend the proposed sensor for nonlaboratory analysis. The correctness of the analysis results has been confirmed by a good correlation with the enzymatic spectrophotometric analysis (R^2^ = 0.9938) and the “added-found” technique (recovery close to 100%).

## Figures and Tables

**Table 1:** Calculated and experimental parameters for voltammograms of UA electrooxidation on macro- and nanostructured electrodes.

No.	Electrode	Calculated		Experimental	
		I, µA	E_1/2_, V	I, µA	E_1/2_, V
1	GCE	0.109	0.49	0.118	0.50
2	Au(20nm)/GCE	0.111	0.47	0.120	0.46
3	Au(5nm)/GCE	0.132	0.42	0.131	0.42
4	Au(14nm)/Au-bulk	0.409	0.46	0.393	0.47

E_1/2_—half-wave potential of UA oxidation.

**Table 2:** Interference of some substances with UA signal (The signal caused by 3 μM UA is taken as 100%).

Interfering Substance	Concentration, µM	Changing Signal
AA	15	+1.3%
L-Triptophan	30	−2.6%
Urea	300	+2.8%
Glucose	300	+4.8%
Creatinine	200	−4.4%

**Table 3:** Analytical characteristics of UA determination with the use of different modified CSPEs.

Electrode	Linear Range, μM	Limit of Detection, μM	Conditions of Signal Formation	pH	Real Sample	Ref.
MWCNTs/CSPE	1–100	0.86	*t*_acc_ = 300 s, open circuit potential LSV	5	urine	[11]
PAA-MWCNTs/CSPE	0.5–30	0.458	*t*_acc_ = 1500 s, open circuit potential DPV	7.5	suppotingelectrolyte	[12]
CS-SWCNTs-IL/CSPE	0.5–1000	0.17	*t*_acc_ = 100 s, *E*_ac c_= −0.1 V LSV	2.4	urine	[13]
Au-nps/CSPE	0.0005–5000	0.0005	FIA, Am	1	suppotingelectrolyte	[16]
UOx-poly(4-ASA)-PB-CSPE	10–200	3	FIA, Am	8.27	urine	[18]
PC–UOx–CA–CoPC–CSPE	15–250	15	ChAm	9.2	urine	[19]
GO/Fe_3_O_4_@SiO_2_/CSPE	0.75–300	0.57	DPV	7	urine	[17]
rGO-CSPE	10–3000	0.35	DPV	7	urine	[14]
β-CD/rGO/CSPE	0.08–150	0.026	DPV	7	blood serum	[15]
2.5% Nf/Au(5nm)/CSPE	0.5–600	0.25	LSV	5	blood serum, milk	This work

Am—amperometry, Au-nps—gold nanoparticles; CA—cellulose acetate, ChAm—chronoamperometry, CoPC—cobalt phthalocyanine, CS—chitosan, DPV—differential pulse voltammetry, *E*_acc_—accumulation potential, FIA—flow injection analysis, GO—graphene oxide, IL—ionic liquid, MWCNTs—multi walled carbon nanotubes, PAA—polyacrylic acid, PB—Prussian blue, PC—polycarbonate, poly(4-ASA)—4-aminosalicylic acid, rGO—reduced graphene oxide, SWCNTs—single walled carbon nanotubes, *t*_acc_—accumulation time, UOx—uricase, β-CD—β-cyclodextrin.

**Table 4:** Analysis results of blood serum samples using 2.5% Nf/Au(5 nm)/CSPE (*n* = 3, *P* = 0.95).

Sample	UA in Serum, mM	UA Added, mM	UA Found, mM (Sample + Additive)	Recovery, %
Serum 1	0.36	0.50	0.91 ± 0.05	109
Serum 2	0.24	0.30	0.57 ± 0.12	110
Serum 3	0.58	0.25	0.84 ± 0.09	103
Serum 4	0.21	0.30	0.54 ± 0.06	110
Serum 5	0.16	0.30	0.49 ± 0.07	107

**Table 5:** Analysis results of milk samples using 2.5% Nf/Au(5 nm)/CSPE (*n* = 3, *p* = 0.95).

Sample	Added, μM	Found, μM	Recovery, %	Sr, %
Sample 1	20.0	20.3	101.5	2.5
Sample 2	20.0	19.9	99.5	1.6
Sample 3	20.0	19.3	96.5	1.9
